# Effect of treadmill exercise on catalepsy and the expression of the BDNF gene in 1-methyl-4-phenyl-1,2,3,6-tetrahydropyridine -induced Parkinson in male *NMRI* mice 

**DOI:** 10.22038/ijbms.2020.37707.8960

**Published:** 2020-04

**Authors:** Neda Nikokalam Nazif, Maryam Khosravi, Ramesh Ahmadi, Maryam Bananej, Ahmad Majd

**Affiliations:** 1 Department of Biology, Faculty of Biological Sciences, North-Tehran Branch, Islamic Azad University, Tehran, Iran; 2 Department of Physiology, Qom Branch, Islamic Azad University, Qom, Iran

**Keywords:** BDNF, Catalepsy, Exercise, IL-10, Parkinson’s disease

## Abstract

**Objective(s):**

It is known that treadmill exercise has beneficial effects on the nervous system. The brain-derived neurotrophic factor (BDNF) plays a role in such effects. This study aimed at investigating effects of intermittent treadmill exercise-induced behavioral, histology, and immunohistochemistry (H&E, TH) measurement of brain interleukin-10 (IL-10) in a mice male model of Parkinson’s disease (PD), which is induced by intraperitoneal injection of 1-methyl-4-phenyl-1,2,3,6-tetrahydropyridine (MPTP), as well as the role of BDNF gene in exercise effects.

**Materials and Methods:**

The animals were divided into Control (C), Saline (S), Parkinson (P), Exercise (E), and Parkinson + Exercise (PE) groups. Bar test was performed for the 21-day protocol with 5 days a week treadmill exercises. In this regard, brains were removed from the skull for H&E, TH, IL-10, and the expression of the BDNF gene using the MPTP male mice PD model.

**Results:**

MPTP reduced the number of DA neurons in the substantia nigra (SNpc), whereas daily exercise administration on 1^st^, 7^th^, 14^th^, and 21^st^ days significantly reduced the catalepsy duration induced by MPTP. The results of H&E and TH studies showed that MPTP significantly reduced the number of TH+ neurons in the SNpc compared with those of the control mice. The MPTP caused a marked decrease in basal protein levels of IL-10 in SNpc and corpus striatum in the Parkinson (P) group as compared with controls. Treatment with Exercise (E) group had the most BDNF expression (3.71), and the Parkinson (P) group also had the least BDNF expression (0.18) relative to controls.

**Conclusion:**

The treadmill exercise having neuroprotective effects in SNpc and corpus striatum has improved MPTP associated with motor deficits. It is considered as a non-pharmacological tool for the management of PD.

## Introduction

The second most common neurodegenerative disorder after Alzheimer’s disease (AD) is Parkinson’s disease (PD), which is characterized by motor dysfunctions such as bradykinesia, tremor, rigidity, and postural instability (1). The loss of dopaminergic (DA) neurons in the substantia nigra pars compacta (SNpc) to the striatum (STR) was confirmed to have an essential role in normal motor function, as the pathological hallmark of PD (2). Researchers indicated that clinical parkinsonian symptoms have emerged when a majority (∼60–70%) of SNpc dopamine neurons (DA) are lost, leading to decreased dopamine levels in the nigrostriatal system (3). There have been various risk factors for sporadic PD, including exposure to pesticides and other toxins, positive family history, and oophorectomy, but age is the most important documented so far (4).It is mentioned that complex aspects of the disease have been long studied on toxin-based animal models for PD (5). The widely-used 1-methyl-4-phenyl-1,2,3,6-tetrahydropyridine (MPTP) mouse model of PD is characterized by substantial gliosis linked to activated astroglia and microglia (6). Researchers have shown that inflammation has an important role in MPTP-induced neurotoxicity (7, 8). These reports indicated that MPTP is oxidized in the brain through a reaction catalyzed with monoamine oxidase B (MAO-B), creating its active form, the ion 1-methyl-4-phenyl-2,3-dihydropyridium (MPP^+^). They indicated that this reaction happens mainly in astrocytes and serotoninergic neurons essentially for the development of MPTP neurodegenerative effects (9). Also, they suggested that DAergic neurons selectively uptake MPP^+ ^through the DA transporter (DAT), where it causes ATP levels to be reduced and oxidative stress to be increased by inhibiting the mitochondrial complex I, culminating in DAergic neuronal death (10).

A study demonstrated that enhancement of neurotrophic factors that promote neurogenesis, decreases inflammation, leading to an improved mitochondrial function. Thus, regular treadmill exercise (TE) results in a protection against neurodegenerative diseases such as PD and AD (11), likely mediated by use-dependent expression of endogenous neurotrophic factors (12,13). Moreover, the conclusion has been similar to the case of human PD so that physical exercise can improve motor behavior and reduce cognitive impairment in MPTP-treated mice (14). Research showed that among the proteins that are putatively involved in the pathogenesis of PD, neurotrophic factors have an important role in the neuroprotection of the dopaminergic phenotype (15). The findings of a study revealed that there is some evidence in the interaction with DA neurons pointing to a specific role of BDNF in the neuronal degeneration observed in PD. It is also proven that decreased BDNF mRNA expression in substantia nigra might play a role in the death of nigral DA neurons that is observed in PD (16). According to the evidence, researchers also proposed that establishment of the proper number of DA neurons in the substantia nigra needs BDNF and that the degeneration of DA neurons in PD may be associated with decreased BDNF biosynthesis. A study indicated that BDNF plays an important role in neuronal differentiation, which is distributed throughout the central nervous system (CNS) and found in hippocampal regions in considerable amounts (17). Also, it exists in the striatum. Thus, it seems to be involved in the survival and maintenance of DA neurons, therefore improving motor performance (18).

 A study showed the scientific evaluation of exercise changing brain-derived neurotrophic factor (BDNF) concentration, which is a key research field in healthy adult populations (19). Additionally, scholars indicated endogenous production of BDNF resulting from voluntary exercise that was shown in adult rats and appears to play an essential role in neuroplastic effects of rehabilitation interventions of humans suffering from neurodegenerative disease. Also, they proved that survival and growth of neurons in pars compacta of substantia nigra and recovery of motor behavior (20, 21) are promoted by BDNF and exercise. Findings showed that systemic administration of MPTP in mice gives rise to the loss of nigrostriatal DA neurons, and is widely used to study the pathophysiological mechanisms underlying DA neuron degeneration in PD (22). Additionally, BDNF might improve neuronal dysfunction and neurodegeneration by modulating 1-methyl-4-phenylpyridinium (MPP^+^)-induced neurotoxicity (23), pathologic brain mitochondria function, or DNA repair through stimulating transcription factors such as CREB (cyclic AMP response element-binding protein) (24, 25). It is said that limiting the development of the inflammatory response is a potential therapeutic strategy for PD (26, 27). It is indicated that through local production of the anti-inflammatory cytokine interleukin-10 (IL-10), it modulates the biological activity of immune cells, thus reducing the production of pro-inflammatory mediators, including cytokines, chemokines, and adhesion molecules and it potently antagonizes the actions of major inflammatory cytokines. A homomeric, pleiotropic cytokine expressed in the CNS by monocytes, astrocytes, and microglia is said to be a biologically active IL-10 protein (28). As well, IL-10 has a short half-life and cannot cross the blood-brain barrier (29); therefore the best way to achieve sustained high expression of inflammatory cytokines within the striatum might be the viral vectors (26). Thus, this study aimed to investigate how 3 weeks of treadmill exercise affect catalepsy, histology, immunohistochemistry of tyrosine hydroxylase, measurement of brain IL-10, and the expression of BDNF gene using the MPTP male mice model of PD.

## Materials and Methods

***Laboratory animals***

 Sixty male *NMRI* mice (n =12; 23–25 g) employed in this study were obtained from the Laboratory Animal of Pasteur Institute. The animals were housed in a plastic acrylic cage at room temperature under standard environment conditions (12 hr light/dark cycle at the temperature of 22-24 ^°^C and humidity of 50%±5%) receiving standard diet and had free access to drinking water. The mice were kept based on standards of animal rights approved by the Islamic Azad University and the Helsinki Treaty. This research was performed by obtaining ethics code approval from the Ethics Committee of the Islamic Azad University^’^s Science and Research Branch.

***Experimental design***

**Figure 1:**
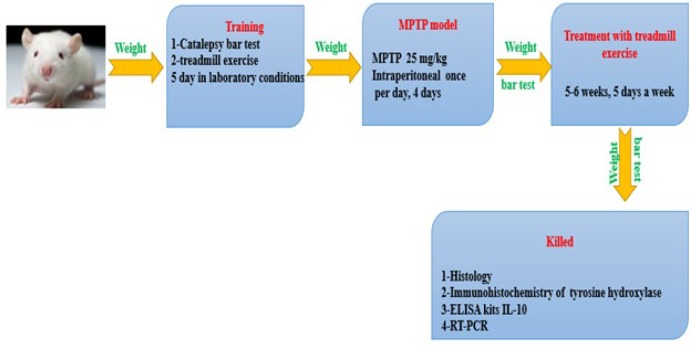
Experimental design

Sixty *NMRI* male mice were divided into five main groups (n=12) for 21 consecutive days:

group 1: Control (C), group 2: Saline (S), group 3: had intraperitoneal administration of MPTP (Parkinson) (P) (25 mg/ kg body weight) for 4 days, groups 4: Exercise (E) (3 weeks, 5 days a week), and group 5: had intraperitoneal administration of MPTP (Parkinson) (P) (25 mg/ kg body weight) for 4 days +Exercise(PE)) (3 weeks, 5 days a week) (Figure 1).

***Chemicals***

1-methyl-4-phenyl-1,2,3,6-tetrahydropyridine hydrochloride, MPTP, was purchased from Sigma-Aldrich Company to be resolved in normal saline 0.9%. MPTP-HCL (25 mg/kg; IP once per day x 4 days), ketamine and xylazine (Sigma), formalin 4% (Sigma), Paraformaldehyde (Sigma), Hematoxylin and Eosin (C5042), Triton X-100 (Abcam), rabbit anti-TH (Abcam), anti-rabbit IgG (Abcam) ELISA kit Interleukin-10 (IL-10) Company abeam. DEPC water, RNA extraction kit (Ribospin ^TM^) (Gene AIL), primer (Macrogene) were the other chemicals used in this study.

***Catalepsy (Bar test)***

The standard bar test was used to determine the intensity of catalepsy (30). Both forelegs of the mice were placed on a horizontal bar (diameter, 0.7 cm) 5 cm above the surface. Animals were placed individually on parallel bars with the forepaws 5 cm above the hind legs and then gently released (on 1^st^, 7^th^, 14^th^, and 21^st^ days; 1 hr post-treatment). The catalepsy duration was recorded in seconds from the moment when an animal was released to the moment when the animal shifted its front paws from the initial position on the upper bar or made gross body or head movements. The trial ended either when a mouse started to move or after 60 sec of immobility (descent latency). The mouse was considered to be cataleptic if the time of immobility was longer than 20 sec.

***Exercise protocol***

After being assigned into five groups (n=12), the exercising groups were trained on the treadmill for 3 weeks. The exercise program involved forced running on the treadmill at the speed of 24 m/min for 3 weeks, 5 days a week, and once a day, each session lasting at least 20–25 min.

***Histologic***

In histology, after the end of the exercise period, the mice using ketamine (50 mg per kg) and xylazine (5 mg per kg) (IP) became insensible, decapitated under deep anesthesia, and their brains were removed from the skull) (n=3 per group) and kept in 4% formalin. After post-fixation overnight, sequential coronal sections 50-μm -thick were made with a freezing microtome, from the level of the SNpc (bregma 2.54 to 3.40 (mm)) according to mouse brain atlas. The sections were stained with Hematoxylin and Eosin (H & E) and immunohistochemistry of tyrosine hydroxylase staining methods. In immunohistochemistry of tyrosine hydroxylase, formalin-fixed and paraffin-embedded sections (5 μm in thickness) received deparaffinization and rehydration treatments. Sections were washed in PBS (0.01 mol/l) three times. Subsequently, the sections were incubated in 3% H_2_O_2_ at room temperature for 10 min. After the blocking step in goat serum for 1 hr, samples were incubated with rabbit polyclonal anti-TH antibody (1:1,000) at 4 ^°^C overnight. Afterward, samples were incubated with biotinylated goat anti-rabbit IgG (1:200) at room temperature for 1 hr followed by incubation in Avidin-Biotin peroxidase Complex (ABC, 1:100) at room temperature for 3 hr. Then, slices were incubated in DAB solution to detect TH protein. Both tissue status and the number of DA neurons in the substantia nigra were counted and analyzed after hematoxylin and Eosin (H & E) and immunohistochemical staining. The section SNpc from brain tissue was imaged using microscope Medacm 107n, the Dino-Lite camera, and Dino Capture 2.0, as well as Image-Pro Plus (V.6) software packages to be evaluated carefully. The present study considered points with a size greater than 7 micrometers as the nucleus of neural neurons.

***Measurement of brain interleukin-10 (IL-10)***

First, mice became insensible using ketamine (50 mg per kg) and xylazine (5 mg per kg), decapitated under deep anesthesia, and their brains were removed from the skull (n=3). In the next stage, the brain tissue was removed to be placed in a refrigerated microtome. Then, this tissue was fixed in a special fixation gel to cut off in 20-micron specimens to the vicinity of the intended area (substantia nigra and corpus striatum). Using the Paxinos & Watson mouse atlas, the cutting was stopped when passing through the olfactory onion to the beginning of the substantia nigra. Herein, corpus striatum and substantia nigra of the brain tissue were punched in two stages using a 1.5-mm diameter punch for the histological evaluation. These specimens were placed separately into the sterilized microtube situating in liquid nitrogen. Following homogenization, samples were shaken (for 90 min) and then centrifuged (at 4 ^°^C, 4000×g, for 15 min) to collect the supernatant. The protein content of the supernatant was examined using a protein assay reagent kit to estimate the amount of protein in both specimens (31). Accordingly, ELISA kits for IL-10 were purchased from the Abcam Company, and assays were performed according to manufacturer’s guidelines.

***Extraction of mRNA***

**Figure 2.**
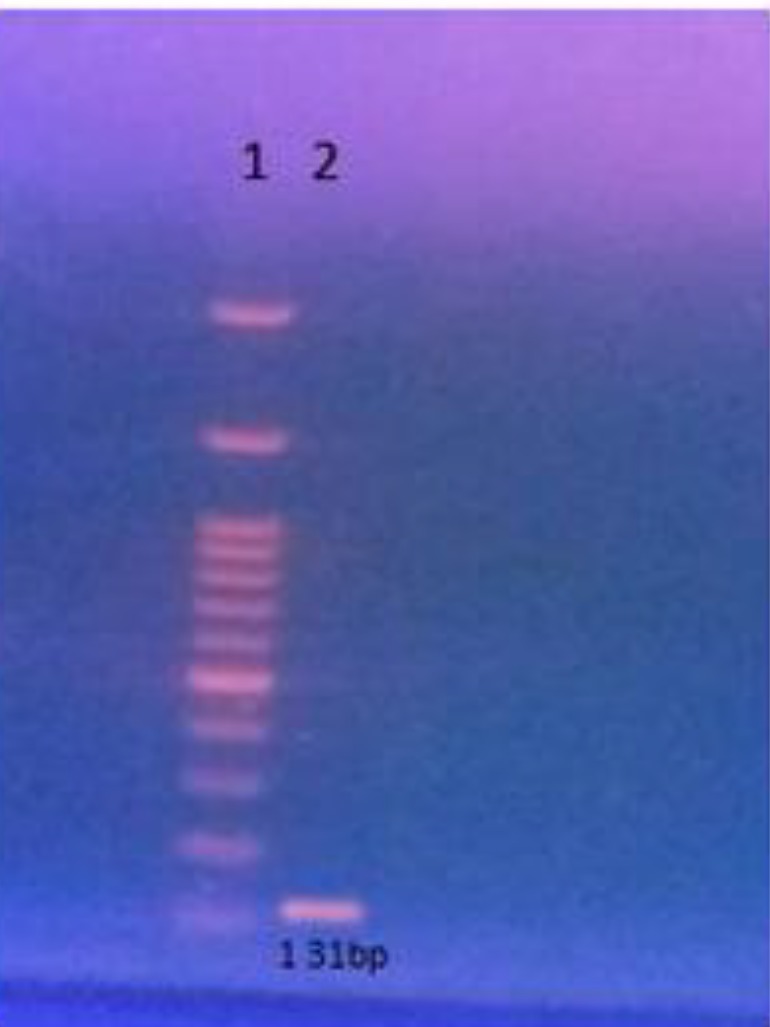
Expression of the BDNF gene in brain. Assays were performed 21 days after inducing the disease with MPTP. 1- marker 100bp, 2-the product PCR gene BDNF

BDNF: brain-derived neurotrophic factor; MPTP: 1-methyl-4-phenyl-1,2,3,6-tetrahydropyridine; PCR: Polymerase chain reaction

For mRNA expression, mice were sacrificed (n=3), and after quick brain removal, SN was dissected on ice and immediately transferred to dry ice to preserve mRNA integrity. Brains were digested in fluid nitrogen. Extraction of RNA and reverse transcription of RNA to cDNA were performed using Gene All Ribospin^TM^ total RNA purification kit (Gene All Biotechnology, Korea) and Gene All hyperscript RT premix kit (Gene All Biotechnology, Korea), respectively, according to manufacturers’ instructions. Briefly, 300 μl of samples were washed twice with phosphate-buffered saline. Then, 0.4 ml of lysis buffer and 10 μl β-mercaptoethanol were pipetted to the sample to lyse the sample through incubation. The lysate was then incubated for 10 min at room temperature and centrifuged to remove drops from inside the lid. The resulting centrifuged mixture was transferred to a mini spin column up to 750 ul. At 25 ^°^C, the mixture was again centrifuged for 30 sec at 10,000 g. Then, the remainder of the sample was examined in the same manner. The mixture containing 500 ul of buffer RBW added to the mini spin column was centrifuged at 10,000 g for 30 sec at room temperature. Once again, 500 ul of buffer RNW was added to the mini spin column, and the solution was centrifuged for 30 sec in 10,000 rpm at 25 ^°^C. Hereafter, 30~50 ul of nuclease-free water was added to the center of the membrane in the mini spin column, and the obtained mixture was centrifuged at 10,000 g for 1 min at room temperature.The ribonucleic acid (RNA) pellet was dried at room temperature for 10 to 20 min and then dissolved in DEPC water remaining at 55 ^°^C for 5 min. The total RNA that had been separated was treated with DNase. RNA 500 ng was added to Gene All hyperscript RT premix kit (Gene All Biotechnology, Korea; Pd No: 601632) to carry out cDNA synthesis for the purpose of using in amplification of the BDNF gene from PCR. Figure 2 shows the BDNF gene expression in the brain.

***Real time-PCR***

Total RNA from the brain was extracted using Ribospin^TM^ (GeneAll) according to the manufacturer’s instructions. RNA was dissolved in nuclease-free water and quantified using spectrophotometry. Then, 1 µg of total RNA was reverse-transcribed to cDNA with Hyperscript^TM^ RT premix with Random Hexamer (GeneAll) according to the manufacturer’s instructions. Primers for real-time PCR of BDNF and GAPDH mRNA were designed according to BDNF and GAPDH mRNA sequences in the mouse. These primer sequences are demonstrated in Table 1. All reactions were conducted following the protocol for the realQ-plus 2x Master mix Green ampliqon. The PCR reaction was performed using a real-time PCR machine (Rotor-Gene 6000) according to the manufacturer’s guidelines. The following PCR protocol was proceeded 40 min to achieve subsequent objectives: denaturation (95 ^°^C for 10 min), amplification (93 ^°^C for 30 sec), and quantification (58 ^°^C for 55 sec) for GAPDH; and denaturation (95 ^°^C for 10 min), amplification (93 ^°^C for 30 sec), and quantification (59 ^°^C for 65 sec) for BDNF; along with a single fluorescence measurement, melting curve (55–95 ^°^C with a heating rate of 1 ^°^C per 30 sec), and the continuous fluorescence measurement. PCR products were identified and distinguished using obtained melting curves. Cycle threshold (Ct) values were defined, representing the cycle number at which sample fluorescence crosses the background statistically. Crossing points (CP) were also determined for each transcript. The relative quantity of gene expression was analyzed by the 2^-∆∆Ct^ method. The quantities of BDNF mRNA were normalized to the endogenous control, GAPDH.

***Data analysis***

Obtained results were expressed through SPSS version 16 using mean±SEM (n=12). The statistical level of significance was determined by one-way ANOVA followed by Tukey’s *post hoc* multiple comparisons test (whichever was applicable). Values of *P≤*0.05 and onwards were considered significant.

## Results

***Catalepsy test***

**Figure 3:**
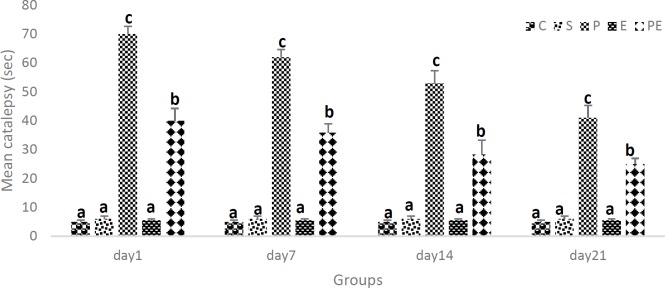
Effect of treadmill exercise on catalepsy a reduced MPTP-induced motor dysfunction as determined in the bar test. Catalepsy days 1, 7, 14 and 21 in groups at Control (C), Saline (S), Parkinson (P), Exercise (E), Parkinson+ Exercise (PE). The mean of catalepsy in Parkinson (P) and Parkinson + Exercise (PE) groups during 21-day significantly reduced (*P*<0.05). The results are shown as mean ± standard deviation (SD); n=12. Dissimilar letters indicate significant differences between the groups (*P*<0.05)

The MPTP administration caused a significant cataleptic effect in mice. Animals’ motor functions were evaluated by the bar test because the loss of DA neurons causes degenerative impairments in motor function. As shown in the previous study, *post hoc* analysis indicated a significant difference in velocity on the 1^st^ day between exercise and MPTP plus exercise groups, whereas no significant difference at the completion of the treadmill running regimen (32). There has also been no significant difference in the mean of catalepsy in Control (C), Saline (S), and Exercise (E) groups over time. While changes in the mean of catalepsy in Parkinson (P) and Parkinson + Exercise (PE) groups during the period of study represented a decrease in the mean of catalepsy over 21 days. In fact, 1 day after the last injection of MPTP, mice showed a significant increase in cataleptic symptoms, which were then reduced on 7^th^, 14^th^, and 21^st^ days. However, the daily exercise administration on 1^st^, 7^th^, 14^th^, and 21^st^ days significantly reduced the catalepsy duration induced by MPTP. The behavioral test revealed that catalepsy test performance was improved in Parkinson + Exercise (PE) mice via treadmill exercise, as well as in MPTP-treated mice with exercise. However, changes observed in the mean of catalepsy in Parkinson (P) and Parkinson + Exercise (PE) groups over the period of the study indicate a decrease in the mean of catalepsy during 21 days (Figure 3).

***Histologic findings***

**Figure 4:**
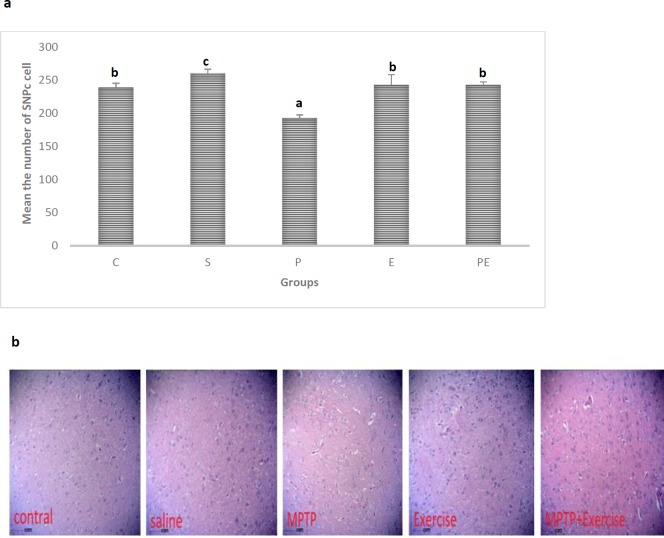
Effect of treadmill exercise on density stained with hematoxylin and eosin (H & E) cells in the SNpc (a). Brain tissues of the region SNpc were analyzed using hematoxylin and eosin stain (magnification, x10) in five groups.Group Parkinson ( P) showed marked changes (*P*<0.05). Parkinson(P) group as compared to Parkinson +Exercise (PE) showed marked changes (*P*<0.05). Dissimilar letters indicate significant differences between the groups (*P*<0.05). (b)Cont; scale bar=50 µm

The results of histological studies showed that the density of Hematoxylin and Eosin cells in the SNpc was significantly decreased in the Parkinson (P) group compared with that in Control (C), Saline (S), Exercise (E), and Parkinson + Exercise (PE) groups *(P<*0.05*)*. There was no significant difference in the mean of the Control (C) group compared with that of Exercise (E), Parkinson+ Exercise (PE) groups *(P>*0.05*). *On the contrary, a significant difference of means was observed for the Parkinson (P) group compared with that for the Control (C), Saline (S), Exercise (E), and Parkinson + Exercise (PE) groups (*P>*0.05)*.* According to the observation, it was suggested that Parkinson + Exercise (PE) might provide neuroprotection by replacing impaired or dead neurons with new neurons in SNpc with an increased number of Hematoxylin - Eosin (H&E) stained neurons in comparison with the Parkinson (P) group. Also, MPTP caused a significant reduction in Hematoxylin - Eosin (H&E) staining of neurons in the SNpc region as compared to the Control (C) group (Figure 4).

***Immunohistochemistry*** ***of tyrosine hydroxylase finding***

**Figure 5:**
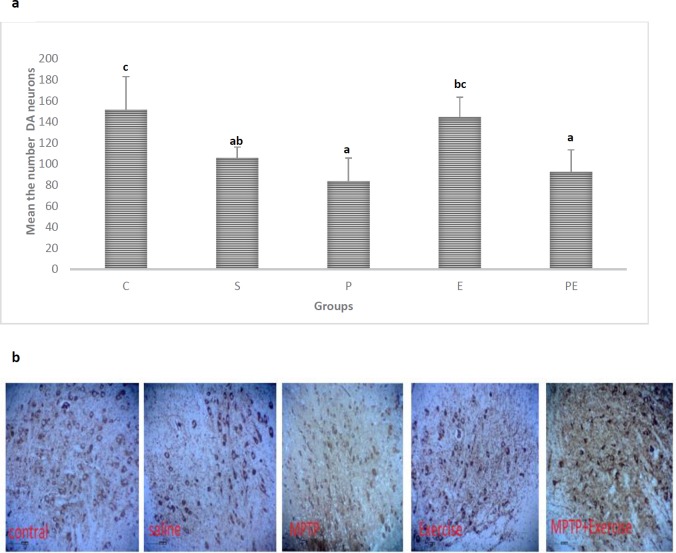
Effect of treadmill exercise on the number of DA neurons stained with immunohistochemistry of tyrosine hydroxylase in the SNpc.(a) .Brain tissues of the region SNpc were analyzed using tyrosine hydroxylase stain (magnification, x10) in five groups. The number of DA neurons in the SNpc Group Parkinson (P) showed marked changes(*P*<0.05), Parkinson(P) group as compared to Parkinson +Exercise (PE) (*P*<0.05) showed marked changes. Dissimilar letters indicate significant differences between the groups (*P*<0.05). (b)Cont; scale bar = 50 µm

Consistent with an earlier work (33), it can be concluded that subacute MPTP injections (25 mg/ kg in 4 days) led to nigral cell loss, as certified by a significant reduction in TH-positive neurons. The number of DA neurons was specified in the SNpc of each treatment group by immunohistochemistry of TH as the rate-limiting enzyme in DA synthesis. These evaluations primarily confirmed and estimated the degree of neuronal loss due to MPTP treatment, but they may also give some insight into whether exercise could influence these changes of structure. The results of immunohistochemistry of tyrosine hydroxylase studies showed a significant reduction in the number of TH^+^ neurons in the SNpc for the treatment with MPTP compared with that of the Control (C) mice (*P*<0.05). Comparison between the treatment groups revealed that numbers of TH^+^ neurons in the SNpc of the mice in Parkinson (P) group without and with Exercise (E) were reduced, compared to Saline (S) and Exercise (E) groups (*P*<0.05) (Figure 5).There was no significant difference in means for Saline (S), Parkinson (P), and Parkinson +Exercise (PE) groups compared with those for Control (C) and Exercise (E) groups *(P>*0.05*)*. However, means for Saline (S), Parkinson (P), and Parkinson + Exercise (PE) groups were significantly different (*P<*0.05). This observation suggested that Parkinson + Exercise (PE) may provide neuroprotection by replacing impaired neurons with new ones in SNpc, with an increased number of TH^+^ neurons in comparison with the Parkinson (P) group. The number of tyrosine hydroxylase in the cell bodies of the substantia nigra was reduced in the MPTP injected mice, on the contrary, treadmill running has enhanced the survival of DA neurons in the substantia nigra (Figure 5).

***Effect of exercise on anti-inflammatory (IL-10) cytokines***

**Figure 6:**
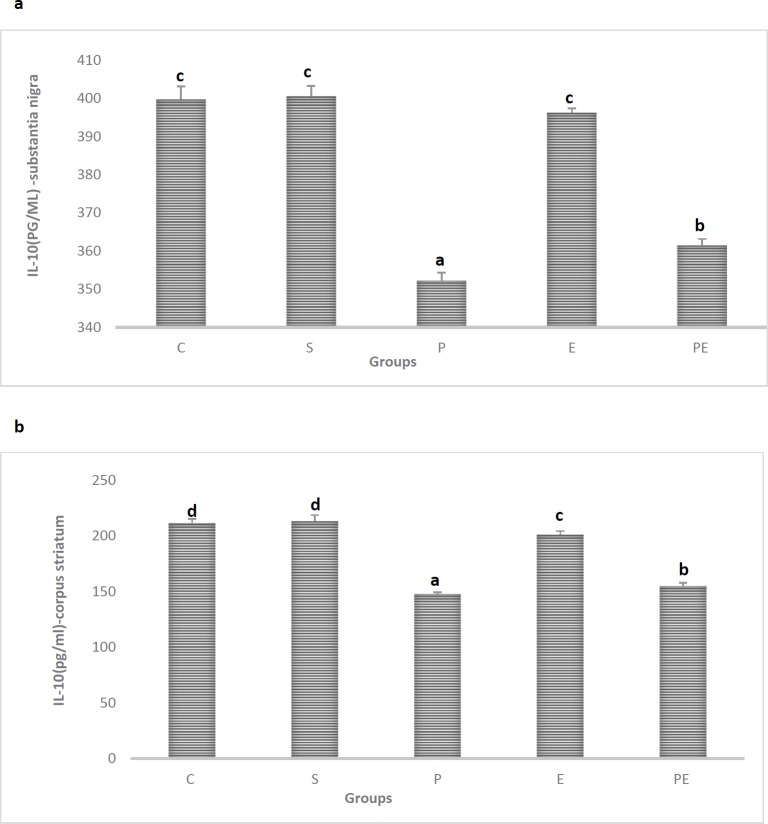
Effect of treadmill exercise on basal protein levels of IL-10 in the substantia nigra (a) and corpus striatum (b) in Control (C), Saline (S), Parkinson (P), Exercise (E), and Parkinson + Exercise (PE) groups. Basal protein levels of IL-10 in substantia nigra (a) and corpus striatum in Parkinson (P) group showed marked decreased (*P*<0.05). Basal protein levels of IL-10 in substantia nigra (a) and corpus striatum in Parkinson + Exercise (PE) group as compared to Parkinson (P) marked increased (*P*<0.05). Dissimilar letters indicate significant differences between the groups (*P*<0.05)

 Recent findings suggested that IL-10 might exert significant neuroprotective effects in MPTP-induced PD through its anti-inflammatory properties (34, 35). The present study evaluated the role of IL-10 in the anti-inflammatory effects of exercise. The MPTP caused a remarkable decrease in basal protein levels of IL-10 in substantia nigra and corpus striatum in the Parkinson (P) group as compared to the Control (C) group *(P<*0.05*)*. There was a significant difference in means for the Parkinson (P) group in the substantia nigra and corpus striatum compared with those in Control (C), Saline (S), Exercise (E), and Parkinson + Exercise (PE) groups *(P>*0.05*)*. IL-10 basal protein levels were increased in mice treated with exercise and decreased in Parkinson +Exercise (PE) groups as compared with that in the Control (C) group. To sum up, MPTP caused a remarkable decrease in basal protein levels of IL-10 in substantia nigra and corpus striatum in the Parkinson (P) group as compared with the Control (C) group Parkinson + Exercise (PE) group ameliorated cytokine levels IL-10 (Figures 6 a, b).

***RT-PCR analysis***

**Figure 7:**
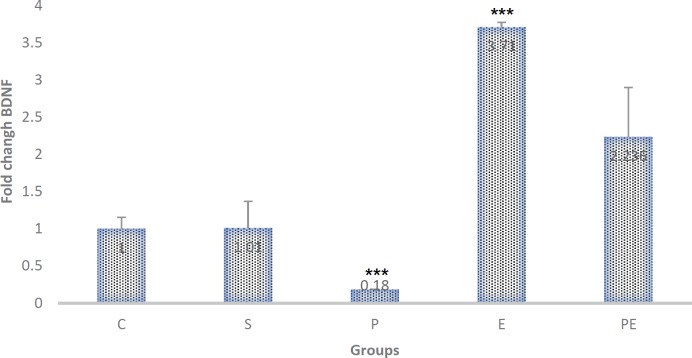
Treadmill exercise BDNF gene expression in mice by MPTP. Mice were treated for 4 days with MPTP, 3 weeks, 5 days a week and once a day. After treatment, mRNA was extracted from each group; quantitative real-time PCR analysis of the BDNF gene was performed. The results are expressed as the fold-change calculated by the relative Ct method using GAPDH as the internal reference. Each bar represents mean±SEM (*P*<0.0001***). Treatment with Exercise (E) group had the most BDNF expression (3.71), and the Parkinson (P) group also had the least BDNF expression (0.18) relative to controls (*P*<0.0001***)

BDNF: brain-derived neurotrophic factor; MPTP: 1-methyl-4-phenyl-1,2,3,6-tetrahydropyridine

Based on the results, the Exercise (E) group had the highest BDNF expression (3.71), and the Parkinson (P) group also had the least BDNF expression (0.18) relative to controls. There was no significant difference of means between Control (C), Saline (S), and Parkinson + Exercise (PE) groups, but means of Parkinson (P) and Exercise (E) groups were significantly different (*P*<0.0001^***^). These results showed that BDNF, in fact, plays a role in neuroprotective effects of exercise (Figure 7).

## Discussion

A loss of DA, which underlies complex structural and functional changes in striatal projection neurons characterizes PD. Interestingly, for SNpc in MPTP-treated animals, loss of DAergic nerve terminals in the striatum precedes the loss of DAergic cell bodies. In this study, the beneficial effects of treadmill exercise on motor functioning and gene expression have been investigated in the MPTP male *NMRI* mice model. The model was validated to investigate the effect of a 3-week treadmill exercise regimen on motor performance, histology, and immunohistochemistry, and evaluation of the level of protein IL-10 and gene expression in BDNF brain areas using behavioral catalepsy test through demonstrating significant nigral neuronal loss following MPTP treatment. 

Previously, the MPTP animal model of PD was shown to suffer from severe motor abnormalities, determined by classical parkinsonian neuro-behavioral phenomena like akinesia and catalepsy (36), as shown in the present study. Researchers reported neuromuscular disabilities in experimental animal models of PD as well as human PD patients (37). Previously, many reports showed that intensive treadmill exercise results in improved motor performance in MPTP-lesioned (32, 38) and saline-treated mice. However, in 2002, a study carried out by researchers indicated increased motor balance and coordination by inhibiting DA neurons and fiber damage in the MPTP mice treadmill exercise. They already knew that exercise could improve motor performance in patients suffering from PD (39). In addition, based on previous studies and recent evidence from animal and human studies, researchers proposed that exercise-induced neurogenesis and neuro-restoration by promoting brain neurotrophic factors, synaptic strength, and angiogenesis may critically lead to regeneration of neurons and thus restore normal motor function (40, 41). In animal studies, exercise has activated the dopaminergic system while enhancing the availability of DA in the striatum (42). Since the death of these neurons interrupts proper communication with cerebral cortex, leading to the impairment in motor function, researchers discussed useful effects of exercise on viability of DA neurons in both striatum and SNpc of MPTP treated mice. In other disease models, treadmill exercise seems to have an effect on ameliorating CNS complications by suppressing apoptotic neuronal cell death and increasing neurotrophic factors (43, 44). Findings indicated that oxidative stress had been involved in the aging brain and most of neurodegenerative diseases, such as PD, AD, Huntington’s disease, and amyotrophic lateral sclerosis (45). Thus, the generalized exercise model presented that the duration is important to protect DA neurons against death, and low-level exercise can partially protect the brain from MPTP-induced free-radical insult (46). In recent studies, researchers have demonstrated the amelioration of cognitive, neurochemical, and mitochondrial function by physical exercise in experimental models (47-49). Accordingly, the study has investigated effects of treadmill exercise on viability of DA neurons in SNpc of MPTP treated mice because the death of these neurons disturbed appropriate relation with cerebral cortex, resulting in disorder in motor function (50).

A study introduced tyrosine hydroxylase (TH) as a rate-limiting enzyme for dopamine synthesis, whereas the dopamine transporter (DAT) is an important determinant of synaptic dopamine concentrations. In PD, reduced expression of TH and DAT results in damaged L-DOPA synthesis and limited neuronal dopamine reuptake, which leads to dopaminergic dysfunction (51) and corresponding motor impairments in PD. In the present study, the number of TH^+ ^cells has significantly been decreased by MPTP treatment in the SNpc (52). Because of the limited sensitivity of mouse plasma dopamine, tyrosine hydroxylase was assessed using immunohistochemistry and treadmill exercise was found to restore the number of TH^+ ^neurons in the SNpc equivalent to controls.

A homodimeric, pleiotropic cytokine originally described as a cytokine inhibitory factor is the anti-inflammatory cytokine, IL-10, by which the actions of the major inflammatory cytokines can be antagonized. Biologically active IL-10 proteins that were expressed in the CNS by monocytes, microglia, and astrocytes have weakened the lipopolysaccharide (LPS)-induced expression of proinflammatory cytokines as well as the induction of neuro apoptosis (53). Similar to PD, MPTP results in multifactorial pathogenesis, among which inflammation has been one of the primary factors (54). However, an investigation performed in 2005 reported that both MPTP and 6-hydroxydopamine (6-OHDA) activate microglia in the SNpc which might precede the death of DA neurons. In the same study, it was shown that anti-inflammatory drugs reducing microglial activation (e.g., minocycline) are somewhat effective in preserving nigral DA neurons. However, the anti-inflammatory action of certain cytokines has not been investigated in these models (55). Anti-inflammatory/regulatory cytokine IL-10, produced by monocytes, but also Th2/Treg cells, was marginally enhanced, possibly as a counter-action towards the high production of inflammatory cytokines, or the change to Th1 (56). According to this concept, the levels of cytokines IL-10 have been measured in the substantia nigra and corpus striatum of PD male mice.

Thus, treadmill exercise has been suggested to have anti-inflammatory influence, leading to neuroprotection in the MPTP male *NMRI* mice model. Nevertheless, researchers reported the anti-inflammatory cytokines, such as transforming growth factors (TGF)-α and -β, and IL-10 in the CNS and serum of PD patients indicating the existence of anti-inflammatory microglia as well (57, 58). On the contrary, Gonzalez *et al*. and Schober *et al.* suggested that cytokines belonging to the TGF-β family and IL-10 are involved in the differentiation and survival of neurons, and exert useful and neuroprotective actions against MPP^+^ toxicity *in vitro* and in PD experimental models *in vivo*. Furthermore, they found that TGF-β co-infusion synergized with glial cell-derived neurotrophic factor (GDNF) has effective impacts on PD models (59, 60). Injection of IL-10 protects DA neurons against LPS-induced cell death (61). IL-10 and TGF-β-induced neuroprotective effects have been suggested to rely, at least in part, on inhibiting cytokine and toxic species production by glial cells (62).

Researchers have shown that the BDNF gene expression is reduced in animal models of PD (63, 64) and humans with PD based on postmortem studies (63). Although this decrease partly results from the loss of DA neurons, the surviving DA neurons in the PD SNpc also show less BDNF (63). Previously, besides abolishing the functional motor and neurochemical (DA) deficits, the exercise-Milmed co-administration was observed to induce also a profound elevation of BDNF levels in the parietal region of the MPTP-treated mice including the motor cortex (65). In particular, according to Parain *et al*. (ref. 66), BDNF was expressed by 65% of the melanized neurons in controls associated with a decreased number of pigmented neurons involving BDNF to 9.6% in the PD SNpc. On the other hand, exercise can restore BDNF levels in animals (67, 68) and parkinsonian patients. *In vitro*, BDNF has neuroprotective influences against the neurotoxicity that is induced by 6-OHDA (69). Some studies also were carried out *in vivo* showing similar results. In view of the useful effects of intermittent treadmill exercise, the BDNF levels were analyzed considering the useful effects of exercise on nervous system. Increasing the BDNF levels in the SNpc of groups with exercise proves that treadmill exercise increases changes in the BDNF levels, activating TrkB-dependent mechanisms related to survival of nigrostriatal DA neurons (64, 70). Instances of such actions include the prevention of apoptosis-mediated cell death and neurotoxin-induced degeneration of DA neurons (71).

BDNF also activates the signaling pathway modulating the transcription factor NFkB, which, in turn, induces the expression of anti-apoptotic proteins and antioxidant enzymes (72). BDNF promotes the survival of DA neurons and protects them from toxin-induced damage *in vitro *(73). A direct nigral infusion of BDNF weakens the decrease in striatal dopamine concentration that is induced by MPTP in mice. The toxic action of systemic MPTP in monkey is reduced by continuous intrathecal injection of BDNF. According to growth factors, previous studies have shown the enhancement of mRNA and protein for these genes following the exercise (74). In the hippocampus, these changes have only been shown to occur when the exercise dynamically continues to perform. The neuroprotective variations in BDNF observed in mice (75) would be a result of BDNF provided through beneficial effects of exercise, as proven in other models (76). In a few studies recently published, it has been demonstrated that the exercise, in part, has neuroprotective impacts on the dopaminergic system and increases the neuronal migration, at least by the modulation of the microenvironment, which includes the up-regulation of BDNF and GDNF. Clinically, based on findings, exercise is indicated to improve gait, mobility, and quality of life of patients with PD, thus reducing the risk of falls. It is clear that BDNF is primarily responsible for survival and difference of DA neurons (77). BDNF is an essential factor in neuronal difference, distributed throughout the CNS and is found in great amounts in hippocampal regions (17). It is shown that, in cases of brain damage, and other functions throughout life, such as learning and memory, BDNF is an important NF in exercise-dependent neuroplasticity and neuron preservation. These functions have been shown in studies that use adult rodents in cell-culture experiments, where upregulation of BDNF promoted the survival of nigrostriatal neurons and other cortical regions. Besides the clinical improvements, physical activity enhances serum BDNF, crossing the blood-brain barrier and may reduce the PD risk (42), illustrating that exercise indeed has neuroprotective effects. Exercises have progressive damage neuroprotective effects, preventing the death of substantia nigra neural cells. Thus, the protective effects of treadmill exercises for 3 weeks, 5 days a week, may increase BDNF gene expression in the brain leading to disease improvement through inhibition of inflammatory pathways that are involved in Parkinson’s disease.

**Table 1:** The sequence of the designed primers used for Real-time polymerase chain reaction

Annealing temperature(°C)	Size (bp**)**	Primer sequences (5′–3′)	Gene
55.91 57.63	131	F5 AAAGCAACAAGTTCCCCAG 3´ R 5´ CCCACTGCTCAGGTCACAC 3´	BDNF
58.34 57.74	222	F 5´AAGGTCATCCCAGAGCTGAA3´ R 5 ´ CCCACTGCTCAGGTCACAC3´	GAPDH

BDNF: brain-derived neurotrophic factor; GAPDH: Glyceraldehyde 3-phosphate dehydrogenase

## Conclusion

The present study examined the anti-inflammatory effects of treadmill exercise for 3 weeks to improve motor function through the protection of nigrostriatal DA neurons as well as increment in basal IL-10 protein levels and BDNF gene expression in the MPTP male NMRI mice model. Based on the results obtained from this study, it can be concluded that treadmill exercise has had a neuroprotective role, improving catalepsy, and increasing anti-inflammatory factors in Parkinson’s disease rodent models. The presented results proposed an exercise-induced increase in BDNF mRNA expression levels in PD.
